# Use of chitin and chitosan to produce new chitooligosaccharides by chitinase Chit42: enzymatic activity and structural basis of protein specificity

**DOI:** 10.1186/s12934-018-0895-x

**Published:** 2018-03-22

**Authors:** Peter Elias Kidibule, Paloma Santos-Moriano, Elena Jiménez-Ortega, Mercedes Ramírez-Escudero, M. Carmen Limón, Miguel Remacha, Francisco José Plou, Julia Sanz-Aparicio, María Fernández-Lobato

**Affiliations:** 1 Department of Molecular Biology, Centre for Molecular Biology Severo Ochoa (CSIC-UAM), University Autonomous from Madrid, C/ Nicolás Cabrera, 1, Cantoblanco, 28049 Madrid, Spain; 2 Institute of Catalysis and Petrochemistry, CSIC, C/ Marie Curie, 2, Cantoblanco, 28049 Madrid, Spain; 3 Department of Crystallography and Structural Biology, Institute of Physical Chemistry Rocasolano (CSIC), C/ Serrano, 119, 28006 Madrid, Spain; 4 Department of Genetic, University of Sevilla, Avenida Reina Mercedes s/n, 41012 Seville, Spain

**Keywords:** Chitinase, *Trichoderma harzianum*, Chit42 3D structure, Chitooligosaccharides, Partially acetylated chitooligosaccharides

## Abstract

**Background:**

Chitinases are ubiquitous enzymes that have gained a recent biotechnological attention due to their ability to transform biological waste from chitin into valued chito-oligomers with wide agricultural, industrial or medical applications. The biological activity of these molecules is related to their size and acetylation degree. Chitinase Chit42 from *Trichoderma harzianum* hydrolyses chitin oligomers with a minimal of three *N*-acetyl-d-glucosamine (GlcNAc) units. Gene *chit42* was previously characterized, and according to its sequence, the encoded protein included in the structural Glycoside Hydrolase family GH18.

**Results:**

Chit42 was expressed in *Pichia pastoris* using fed-batch fermentation to about 3 g/L. Protein heterologously expressed showed similar biochemical properties to those expressed by the natural producer (42 kDa, optima pH 5.5–6.5 and 30–40 °C). In addition to hydrolyse colloidal chitin, this enzyme released reducing sugars from commercial chitosan of different sizes and acetylation degrees. Chit42 hydrolysed colloidal chitin at least 10-times more efficiently (defined by the *k*_cat_/*K*_m_ ratio) than any of the assayed chitosan. Production of partially acetylated chitooligosaccharides was confirmed in reaction mixtures using HPAEC-PAD chromatography and mass spectrometry. Masses corresponding to (d-glucosamine)_1–8_-GlcNAc were identified from the hydrolysis of different substrates. Crystals from Chit42 were grown and the 3D structure determined at 1.8 Å resolution, showing the expected folding described for other GH18 chitinases, and a characteristic groove shaped substrate-binding site, able to accommodate at least six sugar units. Detailed structural analysis allows depicting the features of the Chit42 specificity, and explains the chemical nature of the partially acetylated molecules obtained from analysed substrates.

**Conclusions:**

Chitinase Chit42 was expressed in a heterologous system to levels never before achieved. The enzyme produced small partially acetylated chitooligosaccharides, which have enormous biotechnological potential in medicine and food. Chit42 3D structure was characterized and analysed. Production and understanding of how the enzymes generating bioactive chito-oligomers work is essential for their biotechnological application, and paves the way for future work to take advantage of chitinolytic activities.

**Electronic supplementary material:**

The online version of this article (10.1186/s12934-018-0895-x) contains supplementary material, which is available to authorized users.

## Background

Chitin, a linear polymer of β-1-4 linked *N*-acetyl-β-d-glucosamine (GlcNAc) units, gives strength to the exoskeletons of insects, crustaceans and fungi cell walls, being the most widespread amino polysaccharide in nature. Deacetylation of chitin produces chitosan, polymer containing GlcNAc and d-glucosamine (GlcN) with the latter usually exceeding about 80% of the residues [1]. Chitin and chitosan have been used as functional materials in the fields of food, health or agriculture because of their biocompatibility, non-toxicity and availability from abundant and inexpensive biomass. Poor solubility at neutral pH values of both high molecular-weight biopolymers limits their potential use [2–4], a problem that could be overcome by using their derived oligomers and monomers. Indeed, the chitooligosaccharides (oligosaccharides derived from chitin or chitosan, COS) biological activity is well documented. They showed antioxidant, anti-inflammatory, antimicrobial, antiviral, antihypertensive, anti-tumoral and/or prebiotic properties [3, 5, 6]. The COS properties are strongly dependent on their size (defined by the degree of polymerization, DP) and charge (related to the degree of deacetylation, DD) [7–9]. However, their use is quite limited due to its non-commercial availability.

COS can be produced by enzymatic conversions using chitinases (or chitosanases), chemical methods or by a combination of both strategies employing chitin or chitosan as starting material [10, 11]. Contrary to the chemical hydrolysis that requires extreme reaction conditions of difficult control, the use of enzymes is a more environmentally friendly process that involves softer, specific and controlled conditions [12]. Chitin lytic enzymes are extensively distributed Glycoside Hydrolases (GH), which cleave randomly at internal or terminal end β-1,4 glycosidic linkages of chitin generating COS, di-acetyl chitobiose ((GlcNAc)_2_) and/or GlcNAc units [13–16]. The biotechnological demand for these enzymes grows as the industrial-medical applicability of the products they generate increases. Therefore, to achieve efficient protocols for the production of both, chitinases and the products they generate, constitutes a challenge for the bioconversion of chitin waste.

Family GH18 (http://www.cazy.org) represents an ancient chitinase type found in all kingdoms of life, from lower organisms to humans, and possess a characteristic catalytic module consisting of a (β/α)_8_-TIM-barrel structure [5, 17–19]. All chitinases described in yeast and fungi are included in the family GH18. They are not only involved in exogenous chitin decomposition but also in fungal cell wall degradation and morphogenesis where hydrolytic cleave of chitin is crucial for hyphal growth, septum formation and spore germination [20].

Some strains of the genus *Trichoderma* are used as powerful biocontrol agent against plant pathogens by the production of a wide variety of lytic enzymes, including several chitinases. The *Trichoderma atroviride* P1 strain produces several GH18 chitinases [21, 22]. Among them, the endochitinase Ech42 has been characterized at genomic and protein level and expressed in *Escherichia coli* [23], *Pichia pastoris* [24] and other *T. harzianum* strains [25]. The best heterologous protein level was obtained in *P. pastoris*, ~ 185 mg/L [24]. The role of some conserved residues in the substrate binding and catalysis of this protein has been enlightened using mutational analyses and three-dimensional structural models based on the crystal structure of the chitinase from the pathogenic fungus *Coccidioides immitis* [26, 27].

The chitinase Chit42 from *T. harzianum* (orthologous to Ech42) plays an important role in the fungus anti-phytopathogens activity [28–30]. This protein was able to hydrolyse chitin oligomers with a minimal DP of 3 units [28]. Gen *chit42* has been previously characterized and encodes a protein of 423 amino acids including a putative exportation signal of 34 residues [31]. Transformants of *T. harzianum* overexpressing ~ 20 mg/L of chitinase Chit42 had also been obtained [30].

In this work we have expressed the chitinase Chit42 from *T. harzianum* in *P. pastoris* to ~ 3 g/L, the best level obtained in a heterologous system for this protein. Enzymatic properties of the heterologous protein and its efficiency to produce COS from different chitinolytic materials were evaluated. In addition, crystallographic analysis of Chit42 has been performed to uncover the molecular basis explaining its observed COS-producing specificity.

## Results and discussion

### Cloning and heterologous expression of the Chit42 protein

**Fig. 1:**
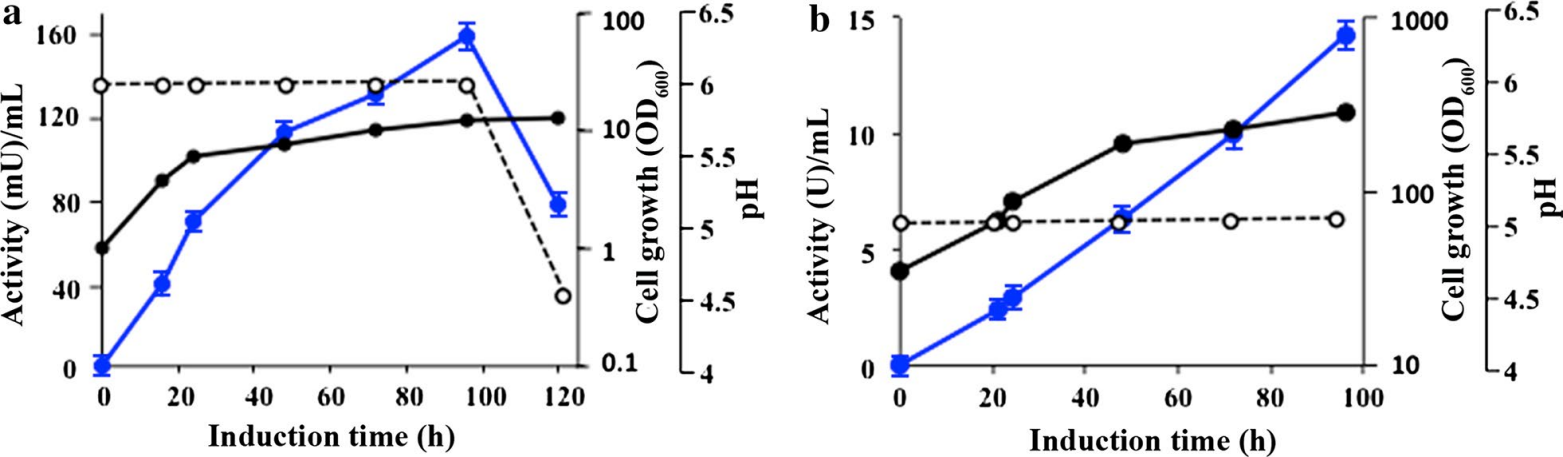
Activity profiles of cultures expressing Chit42. The *P. pastoris* transformant was grown in flask (**a**) and in fed-batch fermenter (**b**) supplemented with methanol. OD_600_ (black circles), pH (empty cycles) and extracellular chitinase activity using colloidal chitin as substrate (blue circles) were measured at the indicated times at 35 °C. Each point of activity represents the average of three independent measurements and standard errors are indicated

**Fig. 2:**
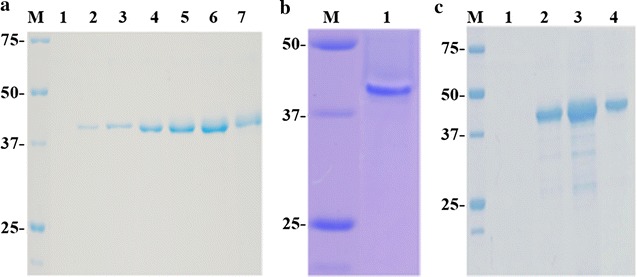
PAGE analyses of Chit42 expressed in *P. pastoris*. Filtrates (5 μL) from yeasts grown in flask were evaluated after 0, 16, 24, 48, 72, 96 and 120 h of methanol induction (lane 1, 2, 3, 4, 5, 6, 7, respectively) using SDS-PAGE (**a**). Filtrate (20 μL) was revealed in situ after 96 h of induction (lane 1) (**b**). Filtrates from yeast grown in fed-batch and induced with methanol during 0, 48 h (0.5 μL; lane 1 and 2), 72 h (0.2 μL; lane 3) and 96 h (0.15 μL; lane 4) were analysed (**c**). Numbers on the left of panels indicate the positions of molecular mass standards (lane M) in kDa

Chitinase Chit42 from *T. harzianum* is an extracellular protein able to hydrolyse chitin oligomers and produce COS with potential biological properties. Overproducing this protein in a heterologous system that allows its future functional improvement is critical for biotechnological application. With this target, the gene *chit42* was included in the *P. pastoris* expression vector pIB4 using a restriction-free cloning strategy. The plasmid CHIT42-pIB4 generated carried the Chit42-expression-cassette flanked by the *AOX1* promoter and terminator sequences. Thus, Chit42 expression was directly controlled by the *AOX1*p and therefore by methanol. In addition, the chitinase signal peptide was replaced by the MFα1 secretion signal, which directed the Chit42 secretion. Transformation of linearized CHIT42-pIB4 into *P. pastoris* gave 21 His + colonies and *chit42* integration into the host genome was confirmed by PCR. The highest chitinase activity, ~ 150 mU/mL culture, was detected in the extracellular medium of one of these transformants grown during 96 h in a methanol based medium (Fig. 1). As expected, this organism secreted into the methanol based medium only a major protein of ~ 42 kDa showing chitinolytic activity (Fig. 2a, b), the same molecular mass than the wild-type protein expressed in *T. harzianum* [28]. No extracellular protein was previously detected in the culture filtrates of control yeasts transformed with the empty vector pIB4 [32]. An extracellular protein concentration of 29 μg/mL was quantified at the point of maximum protein expression, representing a specific chitinase activity of ~ 5.2 U/mg. Production of Chit42 was increased by ~ 100-times, to 2.9 mg/mL (15 U/mL; 5.2 U/mg), by growing the recombinant *P. pastoris* and inducing the protein expression in fed-batch fermentation (Figs. 1b, 2c). As far as we know, this is the highest yield ever reported for the Chit42 from *T. harzianum* expressed in a heterologous system. Expression of fungi chitinases (orthologous to Chit42) in heterologous systems has been previously analyzed with very different results. Thus, only 3 mg/L of chitinase Enc1 from *T. harzianum* T25-1 was obtained in *S. cerevisiae* [33] and less than 200 mg/L of chitinase Ech42 from *T. atroviride* P1 [24] or about 6.2 g/L of chitinase Tachi1 from *T. asperellum* [34], both in *P. pastoris*.

### Biochemical characteristics of Chit42 expressed in *Pichia pastoris*

**Fig. 3:**
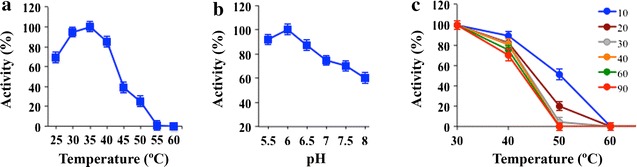
Temperature, pH and thermostability dependence profiles. The effect of temperature (**a**) and pH (**b**) on the Chit42 chitinase activity was evaluated on colloidal chitin at pH 6 and 35 °C, respectively. **c** The chitinase was incubated for the indicated temperatures during the referred time periods (in min) prior to the addition of the substrate. Remaining activity was determined at 35 °C as described in the “Methods” section. Results represent the mean of three independent values. Standard errors are indicated

Heterologous enzyme displayed maximum activity (> 85%) on colloidal chitin at pH 5.5–6.5 and 30–40 °C, and retained less than 40% activity at temperatures above 45 °C. In addition, when enzyme was incubated without substrate in the range of 25–60 °C for 10–90 min and then chitinase activity was assayed, protein maintained 100% activity at 25 °C, completely lost it after 10 min at 60 °C and retained 50% activity in the 43–50 °C range (Fig. 3). Within the conditions tested in this work, the enzyme expressed in *P. pastoris* appeared to be slightly more sensitive to temperature changes than protein previously purified from *T. harzianum* for which maximum activity values were reported at 40–45 °C, and maintained 50% of activity after 30 min at 50 °C [28]. However, our data are closer to what was expected because, in general, the optimal temperature for the fungal chitinase activity varies between 20 and 40 °C [18].

Although less efficiently, chitinase Chit42 expressed in *P. pastoris* was also able to release reducing sugars from commercial chitosan of different sizes and acetylation degrees (Table 1).

**Table 1:** Chit42 hydrolytic activity on the analysed substrates

Substrate	MW (kDa)	DD (%)	Activity (%)
Colloidal Chitin	n.d.	≤ 5^a^	100 ± 6
QS1	98	81	29 ± 2
QS2	31	77	28 ± 2
CHIT600	600–00	> 90	2.3 ± 0.1
CHIT100	100–300	> 90	3.2 ± 0.2

100% activity: 0.2 U/mL

Data are the average of 3 independent experiments. Standard errors were indicated

*n.d.* not determined

^a^DD of initial chitin flakes

Enzyme kinetics with colloidal chitin and chitosan as substrates were examined (Table 2 and Additional file 1: Figure S1), and similar *K*_m_ value for colloidal chitin to that previously reported (~ 1 mg/mL) by using the enzyme expressed in *T. harzianum* [28] was obtained. However, only apparent kinetics parameters were determined using any of the chitosan analysed because its low solubility did not allow a precise estimation of the *V*_max_, and therefore of the *K*_m_ values. Nevertheless, a priori the enzyme showed a very different apparent catalytic efficiency (defined by the *k*_cat_/*K*_m_ ratio) on the tested substrates, and clearly hydrolysed colloidal chitin at least 10-times and 40-times more efficiently than chitosan including a DD in the range of ~ 77–80% and > 90%, respectively (Table 2).

**Table 2:** Catalytic constants on the analysed substrates

Substrate	*K*_m_ (mg/mL)	*k*_cat_ (s^−1^)	*k*_cat_/*K*_m_ (mg^−1^/s^−1^/mL)
Colloidal Chitin	1.7 ± 0.1	5 ± 0.1	3 ± 0.2
QS1	24 ± 12	8 ± 4	0.3 ± 0.2
QS2	2 ± 0.2	0.2 ± 0.02	0.1 ± 0.01
CHIT600	14 ± 4	0.08 ± 0.02	0.006 ± 0.002
CHIT100	9 ± 2	0.07 ± 0.02	0.008 ± 0.002

Apparent *K*_m_ and *k*_cat_ values were obtained using chitosan as substrate. Values of *k*_cat_ were calculated from *V*_max_ considering a Chit42 protein molecular mass of 42 kDa

### Products of the colloidal chitin and chitosan hydrolysis

**Fig. 4:**
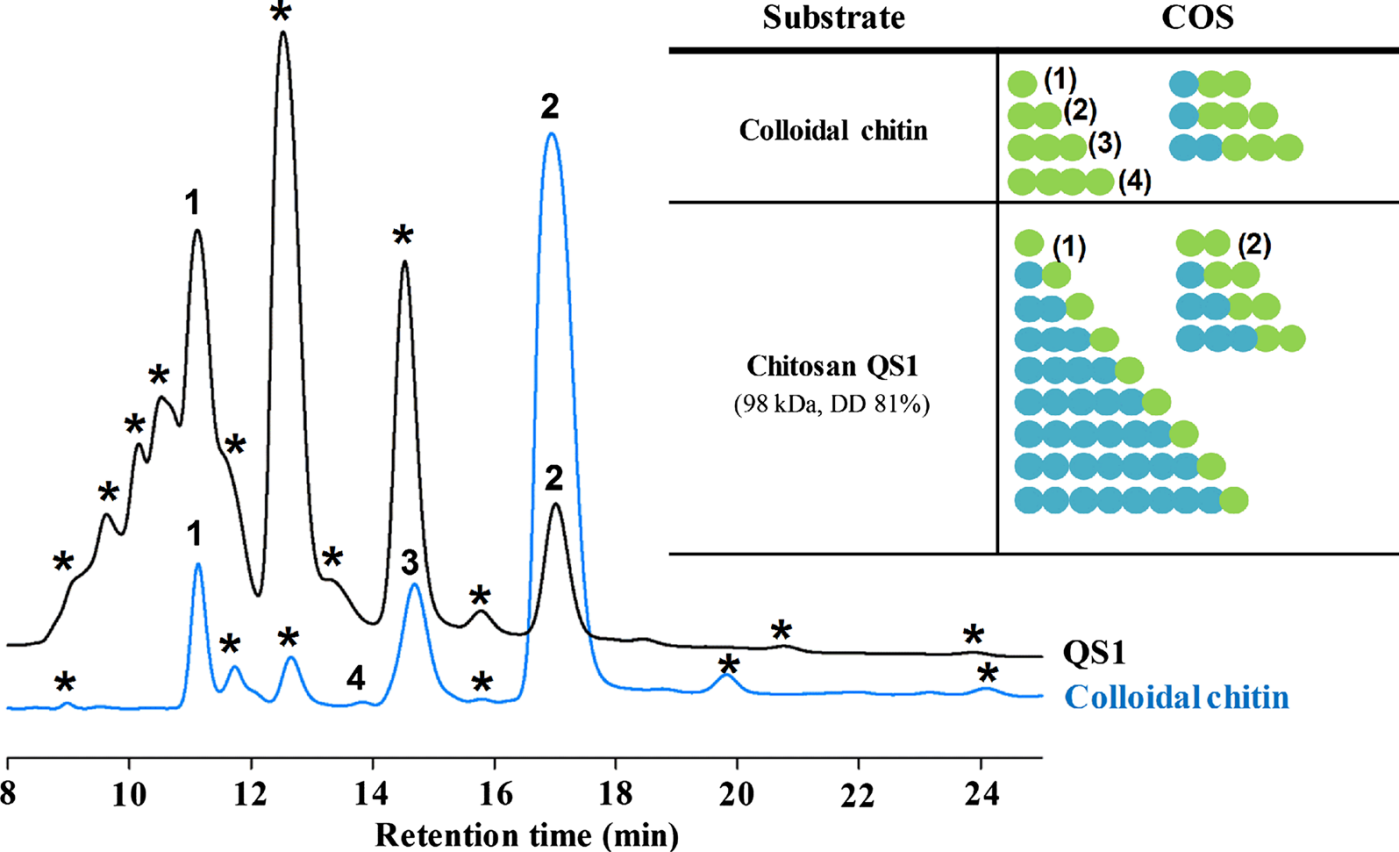
HPAEC-PAD analyses. Chromatograms of the 24 h reactions catalysed by Chit42 with chitosan QS1 (black) and colloidal chitin (blue) as substrates. Peaks: (1) GlcNAc; (2) (GlcNAc)_2_; (3) (GlcNAc)_3_; (4) (GlcNAc)_4_; (*) Unknown. On the right a schematic representation of polymerization degree and composition of reaction products predicted from mass spectrometry data (Additional file 1: Figure S2 and Tables S1, S2) is presented. Blue circles: GlcN. Green circles: GlcNAc. Peaks correspondence in parenthesis

To evaluate the applicability of Chit42 in the COS production, the reaction products using both chitin and chitosan as substrate were analysed by a combination of HPAEC-PAD chromatography and mass spectrometry. With the aid of commercial standards, the fully acetylated series of COS (from 1 to 4 GlcNAc units) was identified when using colloidal chitin as substrate, being the disaccharide the most abundant product (Fig. 4 left, blue chromatogram). Curiously, and although the order of elution with PA-200 columns usually correlates with the increasing DP, the retention time of COS did not follow such order, probably due to the unusual eluting conditions (4 mM NaOH) and that the most acidic hydroxyl group (the 2-OH of glucose moieties) is substituted by NH_2_ or *N*-acetyl. The presence of the commented acetylated oligosaccharides was confirmed in the reaction mixture by mass spectrometry assay (Additional file 1: Figure S2 and Table S1). In addition, and most likely because chitin was not initially 100% acetylated, masses corresponding to partially acetylated COS (paCOS) were also detected in the mixture.

Reactions using chitosan QS1 as substrate (Fig. 4, black chromatogram) yielded numerous signals by HPAEC-PAD, but only two products could be identified: GlcNAc and (GlcNAc)_2_ (peaks 1 and 2, respectively). The remaining peaks did not elute at any known retention time for either fully acetylated or deacetylated available COS. Most likely they must be due to paCOS, as was suggested by the mass spectrometry assay in which masses corresponding to (GlcN)_1–3_-(GlcNAc)_2_ and (GlcN)_1–8_-GlcNAc were detected (Additional file 1: Figure S2 and Table S2). Similar HPAEC-PAD results were obtained using chitosan QS2, CHIT100 and CHIT600 as substrates (Additional file1: Figure S3).

In general, substrate-binding site of fungal chitinases is relatively long and accommodates at least five sugar units. The sugar-binding subsites are denominated as − 3, − 2, − 1, + 1 and + 2, and the cleavage occurring between the − 1 and + 1 sugar. Chitinases from GH18 are retaining enzymes, which means that the β-anomeric configuration found in the substrate is retained in the product, showing an unusual substrate-assisted catalytic mechanism where the acid protonating the glycosidic bond (to be hydrolysed) is a conserved glutamate residue (included in the DXXDXDE sequence), and the nucleophile is the oxygen of the *N*-acetyl group (of GlcNAc) on the subsite − 1 sugar [20]. As described above, Chitinase Chit42 is able to hydrolyse chitin oligomers with a minimal size of 3 GlcNAc units [28] and the catalytic mechanism of chitinases included in the family GH18 requires a mandatory GlcNAc residue in the substrate − 1 position [20]. Thus, based on the enzyme specificity and mass spectrum analyses, it is feasible to think that the highest peak in the HPAEC-PAD chromatogram might well correspond to the tri-saccharide, showing the acetylated residue in the reducing end: (GlcN)_2_-GlcNAc (*N-*acetyl chitotriose). Consequently, this will be very probably the main product obtained from any of the used chitosan.

### Production of COS from different substrates

**Fig. 5:**
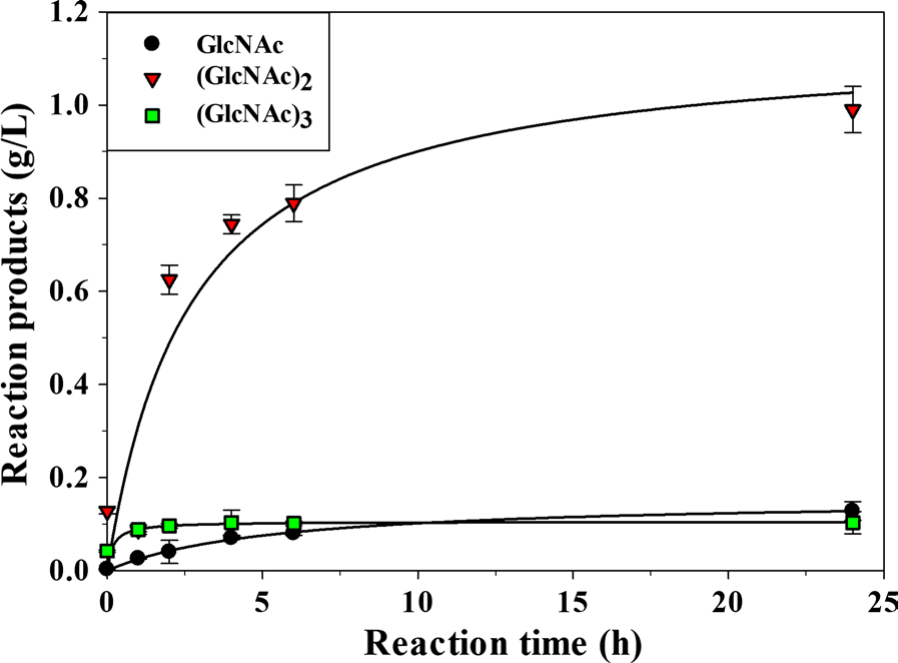
Evolution of the COS produced by Chit42 using colloidal chitin as substrate. Only the identified products (fully acetylated COS with DP 1–3) were quantified and their evolution in the reaction mixtures represented. Each point represents the average of two measurements and standard errors are indicated

Production of COS mediated by Chit42 was evaluated during a total of 24 h using colloidal chitin as substrate. Among other oligosaccharides that could not be characterized by lack of the reference markers, enzyme produced 1.2 g/L of fully acetylated molecules of which 0.13 g/L were GlcNAc, 0.99 g/L (GlcNAc)_2_ and 0.10 g/L (GlcNAc)_3_ (Fig. 5). Standard errors for the quantification of the acetylated COS were lower than 5%. Only small traces of (GlcNAc)_4_ were also detected that could not be quantified (Fig. 4; Additional file 1: Figure S2 and Table S1). Thus, only 15% of the chitin suspension used as substrate was apparently transformed into these small size acetylated COS. Probability that not all the chitin molecules in the initial suspension are in soluble form or even that acetylation degree of colloidal chitin could be different from that of initial chitin flakes, could have contributed to the low COS yield. In fact, the reaction mixtures were quite turbid and all samples precipitated after the NaOH treatment, indicating that they contained polymers of high size that were not hydrolysed.

Concerning the reactions with chitosan as substrate, and because they are mostly deacetylated polymers, a priori, low yields in COS production could be expected. Large zones of substrate lacking GlcNAc residues that the enzyme could not be able to hydrolyse must be taken into account. In addition, as referred, paCOS could not be quantified due to the lack of commercial standards. Thus, only concentration of GlcNAc and (GlcNAc)_2_ could be evaluated in reactions based on chitosan, with values of 0.25 and 0.14 g/L in the case of QS1, indicating that ~ 5% of the substrate was transformed into these two products. A peak with the same retention time as (GlcNAc)_3_ was detected by HPAEC-PAD but the corresponding mass was not detected by mass spectrometry (Additional file 1: Figure S2 and Table S2). Because COS are very difficult to separate, it is not unusual to find two molecules with the same retention time. Lack of (GlcNAc)_3_ in reactions with chitosan can be explained by its high degree of deacetylation, which lowers the probability of finding three consecutive residues of GlcNAc in the chitosan chain. This peak is most likely due to a partially acetylated COS. Also, and in agreement to what was commented above, the major peak in the HPAEC-PAD chromatograms corresponded most likely to the paCOS *N*-acetyl chitotriose.

As referred before, biotechnological applications of COS include anticancer therapy, immune modulatory effect or antioxidant activity among many other [3, 5, 6]. The biological activity of these molecules is related to their DP and DD. Thus, antioxidant activity of paCOS exceeds those of the non-acetylated [9] and small size COS showed stronger antioxidant activity than the bigger ones [35]. In this context, the industrial market demand for COS with defined characteristics increases steadily. The enzymatic synthesis would clearly facilitate the production of homogenous batches of COS with defined properties in comparison to the less specific chitin chemical treatment, which requires large amounts of highly polluting chemical compounds such as HCl and NaOH [36]. All this gives an attractive biotechnological potential to the chitinase Chit42 for the production of small paCOS.

### Structural analysis of Chit42 specificity

**Fig. 6:**
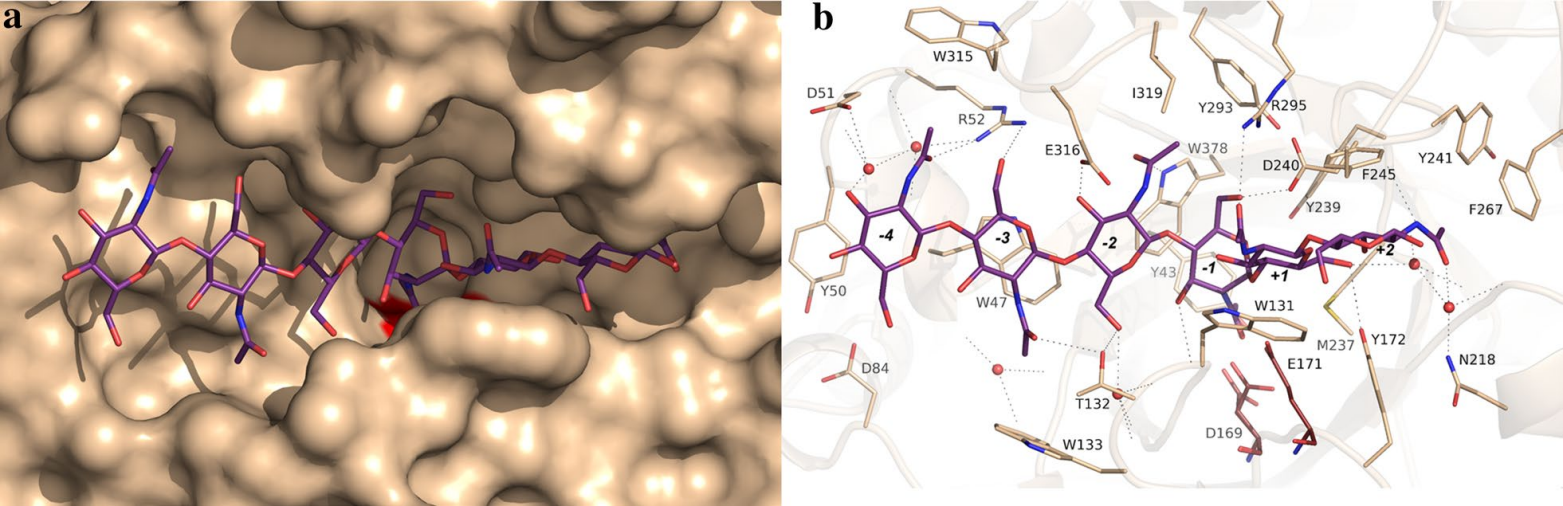
The active site of Chit42. Detail of the crystal structure showing the proposed binding of a COS chain. Sugar was modelled into the active site by structural superimposition with the reported complex from *S. marcescens* ChiA (PDb code 1EIB). Molecular surface of Chit42 showing the sugar in magenta sticks. The catalytic Asp169 and Glu171 are in red (**a**). Details of atomic interaction between COS and Chit42 relevant residues are represented in sticks. Catalytic residues highlighted and proposed hydrogen links represented as dashed lines (**b**)

The structure of Chit42 was solved by molecular replacement at 1.8 Å resolution, showing the structural features previously described for other GH18 chitinases, i.e., a (β/α)_8_ TIM barrel fold with an additional α/β domain inserted within the loop linking helix α8 to strand β9 of the barrel. This extra domain contributes to provide a groove-type shape to the active site (Fig. 6a). We have modeled a chitooligosaccharide within the active site channel by structural superimposition of the reported complex of this substrate with the *Serratia marcescens* ChiA-D313A mutant [37] onto the coordinates of Chit42 here presented. A detail of the proposed interaction with the oligosaccharide is shown in Fig. 6b. Although ChiA and Chit42 sequences are only 26% similar, the corresponding residues located at the active site are rather conserved. Therefore, the distorted conformation of the substrate at the cleavage point observed for ChiA fits well within the Chit42 active site groove. Thus, the complex model illustrates the main features involved in binding. First of all, the substrate is tightly recognized at subsites − 4 to + 2 by a net of hydrogen links to several Chit42 residues. Therefore, there are at least 6 substrate-binding subsites in this protein. In particular, it seems that all the acetyl groups may be involved in hydrogen links with the expected exception of the sugar located at subsite − 1. In general, each sugar makes two hydrogen links, one through the acetyl and the other through the O3/O6 hydroxyls, but the sugar at subsite − 2 seems able to interact through all its free oxygen atoms.

The flexibility of the different subsites in allocating deacetylated COS within a *Trichoderma* chitinase has been discussed before [26, 27]. Taking into account these studies, it was previously proposed that subsites − 2 to + 2 are more specific for GlcNAc binding, while distal subsites at the non-reducing end can accommodate modified sugars. However, and according to the analysis here reported, masses of the paCOS corresponding to (GlcN)_1–3_-(GlcNAc)_2_ and also to (GlcN)_1–8_-GlcNAc, have been identified from the hydrolysis of chitosan QS1 (Additional file 1: Figure S2 and Table S2). Therefore, the acetyl moiety at subsites -2 must not be strictly required. Previous mutagenesis experiments revealed that polar interaction of substrate with the *Trichoderma* chitinase residue Glu316 at subsites − 3 and − 2, is essential in binding [27]. According to our complex model, Glu316 is hydrogen linked to O3 of the sugar at subsite − 2, but seems also able to link to a free amine group by a slight switch of its side-chain, therefore compensating the loss of the hydrogen bond to Trp378 and contributing to stabilized a deacetylated sugar at this subsite − 2. With respect to the sugar located at subsite − 3, the stacking interaction to Trp47 was previously proved to be critical in substrate binding, therefore making a broad specific subsite able to allocate modified sugars. Thus, a galactose unit, or fucose as a branch, were both accepted at subsite − 3 [26]. According to our model, Arg52 is also involved in allocating the sugar at this subsite; therefore, its flexible chain provides additional plasticity to the sugar type occupying this binding subsite. Consequently, the structural analysis sheds light on the Chit42 specificity observed in our work, and explains the chemical nature of the partially acetylated products obtained from chitosan.

A last interesting feature is the fact that the side-chain of Trp131, making subsite + 1, presents rather weak electron density in our solved free enzyme crystal, revealing a marked disorder. This observation might be indicative of a process mechanism as proposed for the *S. marcescens* ChiA, where mobility of this aromatic residue may be essential to perform a productive sliding of the substrate. Alternatively, flexibility of this Trp131 might be required to facilitate distortion of the sugar occupying subsite − 1. In agreement with the disorder observed by us, two alternate conformations were previously found for this conserved Trp in the un-complexed crystals grown from the *Aspergillus fumigatus* ChiB1 [38]. Our work provides useful information on the functionality of Chit42, a chitinase of biotechnological interest for the production of pCOS, and could be useful to understand the enzymatic behaviour of other proteins included in the GH18 family.

## Conclusion

Chitooligosaccharides have enormous biotechnological potential in medicine and food. Production and understanding of how the enzymes that generate them work is essential for their biotechnological production and application. Chitinase Chit42 has been overexpressed in a heterologous system to levels never before achieved and its activity on different chitinolytic substrates tested. The enzyme produces partially acetylated chitooligosaccharides, which confers it biotechnological interest to obtain high value products from the waste of industrial activity. The presented structural analysis provides the molecular basis for understanding protein product specificity, and paves the way for future work to take advantage of Chit42 activity.

## Methods

### Chemicals

Chitin (from shrimp shells, practical grade coarse flakes; DD ≤ 5%), glycol chitosan, *N*-acetyl-glucosamine (GlcNAc) and Biotin were from Sigma Aldrich (St. Louis, MO, USA). Colloidal chitin was obtained from chitin by the method of Jeuniaux [39]. Basically, 175 mL of 10 M HCl including 10 g of chitin was maintained 16 h at 4 °C and filtered through glass thick fibers into 1 L of ethanol. Chitin floccules were precipitated after 16 h at 4 °C, collected at 5000×*g* during 10 min and washed with distilled water. Then, 200 mL of 70 mM potassium phosphate pH 6 was added and colloidal chitin concentration was estimated by titrating the weight of solute contained in 1 mL of solution that was previously frozen at − 70 °C and lyophilized.

Glycol chitin was obtained from glycol chitosan as previously reported [9]. Basically, 0.2 g of glycol chitosan was suspended in 50 mL methanol 50% (v/v) and 0.3 mL acetic anhydride. Two volumes of acetone were added and sample was centrifuged at 5000×*g* during 10 min. Precipitate was treated with 1 M sodium hydroxide, dialyzed against water, frozen at − 70 °C, and lyophilized. Chitosan CHIT100 and CHIT600 were from Acros Organics (Thermo Fischer Scientific Inc., Waltham, MA). Chitosan QS1 (from *Paralomis granulosa*) and QS2 (from *Pandalus borealis*) were from InFiQuS (Madrid, Spain). Chitosans (1 g) were dissolved in 90 mL of 0.1 M acetic acid and then 10 mL of 1 M sodium acetate pH 5.5 was added (1% (w/v) chitosan final concentration in 100 mM sodium acetate pH 5.5). Chitobiose ((GlcN)_2_), chitotriose ((GlcN)_3_), chitotetraose ((GlcN)_4_) and *N*,*N*′,*N*ʺ-tri-*N*-acetyl-glucosamine ((GlcNAc)_3_) were from Carbosynth Ltd. (Berkshire, UK). Yeast Nitrogen base w/o amino acids (YNB) was from Difco (BD, Sparks, MD, USA). All other reagents were of the highest purity grade.

### Strains, growth and expression media

*Pichia pastoris* GS115 (*his4*-) (Invitrogen, Carlsbad, CA, USA) was used as expression host and was initially cultured at 30 °C and 250 rpm shaking in YED (1% yeast extract, 1% peptone, 2% glucose; all w/v). The yeast transformants were selected on MD medium (13.4 mg/mL YNB, 4 mg/mL biotin, 2% glucose; all w/v). Expression of the Chit42 protein was analysed on BMM after growing in BMG (both media same as MD but in 100 mM potassium phosphate pH 6.0 and 0.5% methanol or 1% glycerol as carbon source, respectively). BMG-F medium (same as BMG but 100 mM potassium phosphate pH 5.0 and 4% glycerol) was used for *P. pastoris* growth to high cell density. Growth was monitored spectrophotometrically at a wavelength of 600 nm (OD_600_). The *Escherichia coli* DH5α strain was used as host for DNA manipulations using the standard techniques.

### DNA amplification and cloning

The chitinase *chit42* cDNA from *T. harzianum* CECT2413 comprised of 1272 bp (GenBank accession no. S78423.1), which codes for a protein of 423 amino acids (P48827), with a signal peptide of 34 residues, and was previously included in plasmid pCHIT42, a pBluescript SK (+) derivative [31]. In this work, plasmid CHIT42-pIB4, a derivative of the pIB4 (*His4*) vector including the methanol-regulated alcohol oxidase promoter (*AOX1*p) of *P. pastoris* [40], was obtained to express Chit42 fused to the *Saccharomyces cerevisiae* MFα1 secretion signal in *P. pastoris*. For that, the restriction-free cloning strategy reported by Van den Ent and Löwe [41] was used. Basically, a PCR fragment containing the gene of interest (*chit42* cDNA fused to short sequences which are complementary to sequences flanking the site of insertion in the receptor vector) was used as a pair of primers in a linear amplification reaction around a circular plasmid acting as a template (plasmid QDNS-pIB4). Thus, the gene *chit42* was amplified from construction pCHIT42 using primers: CHIT42F: 5′-gagaaaagagaggctgaagctGCCAACGGATACGCAAACTC-3′ (MFα signal peptide sequence in lower case) and CHIT42R: 5′-actgaggaacagtcatgtctaagaagcttCTAGTTCAGACCATTCTTGATGTTATCA-3′ (pIB4 sequence in lower case). Phusion High-fidelity DNA polymerase (NEB, Ipswich, UK) was used with the following conditions of amplification: (i) 98 °C for 30 s; (ii) 25 cycles of 98 °C for 10 s, 55 °C for 30 s and 72 °C for 40 s; (iii) final extension at 72 °C for 600 s. The PCR product (1220 bp) was purified from agarose gel using Wizard SV Gel kit (Promega, Madison, USA) and used as primer in a second PCR reaction where plasmid QDNs-pIB4 was the template. This, pIB4 derivative plasmid had previously been used to express the β-fructofuranosidase *Xd*-*INV* gene from the *Xanthophyllomyces dendrorhous* yeast in *P. pastoris* and included the last 1902 bp of *Xd*-*INV* fused to the 267 bp fragment of the MFα1 secretion signal [32]. Conditions of amplification were: (i) 98 °C for 30 s; (ii) 35 cycles of 98 °C for 10 s, 55 °C for 30 s and 72 °C for 240 s; (iii) 72 °C for 600 s. Then, PCR reaction mixture was treated with *Dpn*I to digest the methylated template, and then transformed into *E. coli* cells. Colonies including the generated CHIT42-pIB4 plasmid were detected by PCR using primers: AOX1: 5′-GACTGGTTCCAATTGACAAGC-3′ and AOX2: 5′-CCTACAGTCTTACGGTAAACG-3′, both from Sigma Aldrich (St. Louis, MO) and directed to sequences in the vector flanking the site of insertion, with generate a 1527 bp amplification product. In the CHIT42-pIB4 construction, the 1902 bp of the gene *Xd*-*INV* was cleanly replaced by the last 1167 bp of *chit42*, which was fused to the MFα1 secretion signal sequence that includes the ATG initiation triplet. In addition, expression of Chit42 was under the control of *AOX1*p, which means that protein production can be strongly induced by methanol. Integrity of CHIT42-pIB4 construction was verified by DNA sequencing.

### *Pichia pastoris* transformation and protein expression

Plasmid CHIT42-pIB4 (6 μg) was linearized with *Stu*1 (into *His4*) and transformed into *P. pastoris* by electroporation according to the manual for protein expression in *Pichia* (Invitrogen, Carlsbad, CA, USA). Integration of gene *chit42* in the transformants genome was confirmed by PCR using the previously referred primers CHIT42F and CHIT42R. Transformants including the empty vector pIB4 were also obtained and used as controls. Expression of chitinase Chit42 in *P. pastoris* was analysed using BMM medium and heterologous activity was evaluated by measuring chitinase activity in culture filtrates. Initially, transformants carrying the construction CHIT42-pIB4 were grown at 30 °C in 25 mL of BMG during 24 h, with shaking at 250 rpm, and then in 200 mL of BMM using 1 L flasks. Both, yeast growth (OD_600_) and the pH of the cultures were evaluated. Cells were removed at 6000×*g* for 15 min. Extracellular fraction was concentrated and fractionated (if required) trough 30,000 MWCO PES membranes by using a Vivaflow 50 system (Sartorius, Gottingen, Germany). About 68% of the chitinase activity was recovered. Yeast Protein concentration was determined in a NanoDrop 1000 Spectrophotometer, V3.8 Thermo Fisher Scientific Inc (Wilmington, USA) at 280 nm using bovine serum albumin as standard.

### Fed-batch fermentation

Recombinant *P. pastoris* expressing Chit42 was cultivated in 500 mL of BMG-F medium (three 1-L flasks containing 166 mL of BMG-F each) during 24 h and then cultivated to high cell density fed-batch fermentation using a 5-L bioreactor (Biostart BPluss Sartorius Ltd., Gottingen, Germany) containing 3.5 L of a batch medium including per 1 L: 40 g glycerol, 26.7 mL H_3_PO_4_ 85%, 0.93 g CaSO_4_, 18.2 g K_2_SO_4_, 14.9 g MgSO_4_·7H_2_O, 4.13 g KOH, 2 mL biotin (0.2 g/L) and 4.35 mL of PTM_1_ trace salts (Invitrogen, Carlsbad, CA, USA). Initial OD_600_ of the culture was ~ 0.28 units. The fermentation parameters were maintained at 30 °C, 600 rpm agitation, 20% dissolved O_2_ and pH was controlled at 5.0 with NH_4_OH 28% (v/v) during 24 h (~ 40 OD_600_). Then 100% methanol was added continuously during 4 days at a rate of 20 μL/min/L of fermentation volume to induce the expression of protein Chit42 (final OD_600 _~ 290 units). Chitinase activity and protein concentration of the fermenter culture were monitored throughout the process. Protein concentration was determined using NanoDrop at 280 nm as referred above.

### Enzyme and kinetic analysis

Unless otherwise indicated, chitinase activity was determined by detection of reducing sugars from colloidal chitin. Reactions were performed in 1.5 mL Eppendorf tubes by addition of 100 µL of the enzymatic solution (previously diluted in 70 mM potassium phosphate pH 6, if required) to 400 µL of 1% (w/v) colloidal chitin and other substrates. Tubes were incubated at 900 rpm in a Thermo Shaker TS-100 (Boeco, Hamburg, Germany) during 30 min. A range of 25–60 °C was used in the temperature dependence activity assay. Reactions were boiled for 10 min at 100 °C and one volume of 0.2 M NaOH (for precipitation of the remaining polysaccharides) was added. Polysaccharides were removed by centrifugation at 12000×*g* for 5 min. The quantification of reducing sugars in the supernatant was carried out using 3,5-dinitrosalicylic acid (DNS) method adapted to a 96-well microplate scale as described elsewhere [42]. A calibration curve of d-glucosamine (0–3 mg/mL) was used. One unit of chitinase activity (U) was defined as that corresponding to the release of 1 μmol of reducing sugar per minute.

For estimation of chitinase activity at different pH values colloidal chitin was used in 70 mM potassium phosphate at the pH range: 5.5–8.0 and used as referred above. Unless otherwise indicated, activity was tested at 35 °C. The thermostability refers to the temperature required for 50% activity inactivation after maintaining the enzyme at 43–50 °C during 10–90 min, removing samples at regular intervals and estimating the residual chitinase activity. All the reactions were performed in triplicate. The Michaelis–Menten kinetic constants were determined using 0.1–15 mg/mL of analysed substrates and 35 °C. The plotting and analysis of the curves was carried out using SigmaPlot software (version 11.0), and the kinetic parameters were calculated fitting the initial rate values to the Michaelis–Menten equation. Standard errors were obtained by fitting the normalized equation as v = (*k*_cat_/*K*_m_)[S]/(1 + [S]/*K*_m_).

### SDS-PAGE and zymogram analyses

InstantBlue protein Stain (Expedeon, Cambridge, UK)-sodium dodecyl sulphate–polyacrylamide gel electrophoresis (SDS-PAGE 12%) of samples confirmed their protein level. Gels were prepared and processes according to the standard Laemmli method [43]. Precision Plus Protein Standards Unstained 10–250 kDa (Bio-Rad, CA, USA) were used as weight markers.

Chitinolytic activity was detected by zymogram analysis using basically the methodology developed by Zur et al. [44]. Proteins were separated on non-denaturing gels (PAGE 12% without SDS) containing 0.1% (w/v) glycol chitin. Gels were run at 4 °C at 180 V. After electrophoresis, gels were soaked in 100 mM sodium acetate pH 5.5 containing 1% (v/v) Triton X-100 and incubated with gentle agitation for 15 min at room temperature. Then, 100 mM sodium acetate pH 5.5 was added and gels were incubated during 1 h at 35 °C for the in gel-chitinolytic reaction. Finally, gels were washed with distilled water during 5 min, stained with 2.5 mg/mL Coomassie Brilliant Blue R-250 (Bio-Rad, CA, USA) during 20 min, and then 20% (v/v) acetic acid was added. Chitinolytic activity was detected as a clear area (halo) against a dark purple background.

### Characterization, quantification of COS by HPAEC-PAD and mass spectrometry

Reactions were performed as described above using ~ 0.2 units of chitinase activity/mL of assay. Aliquots of 0.2 mL were withdrawn at different reaction times, mixed with 0.2 M NaOH and centrifuged as referred. The supernatant was diluted with 2.5 mM NaOH (final concentration) and analysed by HPAEC-PAD as described before [45]. The chromatography equipment was a Dionex ICS3000 system (Dionex, Thermo Fischer Scientific Inc., Waltham, MA) consisting of an SP gradient pump, an electrochemical detector with a gold working electrode and a Ag/AgCl as reference electrode, and an auto sampler (model AS-HV). An anion-exchange 4 × 250 mm Carbo-Pack PA-200 column (Dionex) connected to a 4 × 50 mm CarboPac PA-200 guard column was used at 30 °C. The initial mobile phase was 4 mM NaOH at 0.3 mL/min for 30 min. Then, column was washed for 20 min at 0.5 mL/min with a solution containing 100 mM sodium acetate and 100 mM NaOH, and further equilibrated with 4 mM NaOH. Standards of fully deacetylated COS with DP ranging from 1 to 5 and fully acetylated COS with DP 1–4 were used to build the calibration curves for HPAEC-PAD analysis. Individual compounds were dissolved in NaOH (final concentration 10 mM) and serial dilutions were made from 0.12 to 0.005 g/L. Curves were adjusted to cubic or quadratic regressions using Chromeleon Software.

The molecular weight of COS was assessed by MALDI-MS using a mass spectrometer with Ultraflex III TOF/TOF (Bruker, Billerica, MA, USA) and an NdYAG laser. Registers were taken in positive reflector mode within the mass interval 40–5000 Da, with external calibration and with 20 mg/mL 2,5-dihydroxybenzoic in acetonitrile (3:7) (v/v) as matrix. Samples were mixed with the matrix in a 4:1 proportion and 0.5 µL were analysed.

### Crystallization, data collection and crystal structure determination

Initial crystallization conditions for Chit42 (28 mg/mL) were explored by high-throughput techniques with a NanoDrop robot (Innovadyne Technologies Inc.), using four different commercially screens: PACT and JCSG + Suites from Qiagen; and Index and SaltRx packages from Hampton Research. Assays were carried out using the sitting-drop vapour-diffusion method in MRC 96 well crystallization plates (Molecular Dimensions). Elongated twinned needles grew from 20% polyethylene glycol (PEG) 3000, 0.2 M zinc acetate, 0.1 M imidazole pH 8, from JCSG crystallization screen. Conditions were further optimized by diluting the protein to half concentration (14 mg/mL) and including micro seeds in the drops, to final conditions containing 22% PEG 3K, 0.1 M zinc acetate, 0.1 M imidazole pH 8. For data collection, crystals were cryoprotected in mother liquor supplemented with 20% (v/v) ethylene glycol before being cooled in liquid nitrogen.

Diffraction data were collected at the ALBA synchrotron station of Barcelona, Spain. Diffraction images were processed with XDS [46] and scaled using Aimless from the CCP4 package [47] leading to space group P4_1_2_1_2. The structure was solved by molecular replacement using MOLREP [48] with reflections up to 2.0 Å resolution range and a Patterson radius of 40.8 Å. The template model was the chitinase from *Clonostachys rosea* (PDB code 3G6L). Preliminary rigid-body refinement was carried out using REFMAC [49]. Subsequently, several rounds of extensive model building with COOT [50] combined with automatic restrain refinement with flat bulk solvent correction and using maximum likelihood target features, led to a model covering residues Ala35-Asn423. At the latter stages, imidazole, ethylene glycol, acetate and Zn ions and water molecules were included in the model, which, combined with more rounds of restrained refinement, led to a final R-factor of 18.6 (Rfree 22.1). The free R-factor was calculated using a subset of 5% randomly selected structure-factor amplitudes that were excluded from automated refinement (final refinement parameters: Table 3). The figures were generated with PyMOL [51], and the atomic coordinates have been deposited in the RCSB Protein Data Bank under the accession code 6EPB. The active site contains acetate and Zn tightly bound at the active site, both ions being required for crystal growth. This fact impeded getting complexes by, either crystallization or soaking and, therefore, a complex was modelled as explained in the “Results and discussion” section.

**Table 3:** Crystallographic data of Chit42

Crystal data	
Space group	*P*4_1_2_1_2
Unit cell parameters (Å)	
a	68.35
b	68.35
c	178.28
Data collection	
Beamline	XALOC (ALBA)
Temperature (K)	100
Wavelength (Å)	0.9792
Resolution (Å)	68.35–1.75 (1.75–1.78)
Data processing	
Total reflections	276,828 (15328)
Unique reflections	43,534 (2325)
Multiplicity	6.6 (6.4)
Completeness (%)	99.8 (99.3)
Mean *I*/σ (*I*)	16.9 (3.6)
*R*_*merge*_^a^ (%)	7.4 (60.1)
*R*_*pim*_^b^ (%)	3.2 (24.9)
Molecules/ASU	1
Refinement	
R_work_/R_free_^c^ (%)	18.58/22.11
Nº of atoms/average B (Å^2^)	
Protein	3033/19.51
Other molecules	108/38.65
Water molecules	313/29.55
All atoms	3454/21.02
Ramachandran plot (%)	
Favoured	96
Outliers	0
RMS deviations	
Bonds (Å)	0.009
Angles (°)	1.322
PDB code	6EPB

Values in parentheses are for the high-resolution shell

^a^R_merge_ = ∑_hkl_ ∑_i_ | I_i_(hkl) − [I(hkl)]|/∑_hkl_ ∑_i_ I_i_(hkl), where I_i_(hkl) is the ith measurement of reflection hkl and [I(hkl)] is the weighted mean of all measurements

^b^R_pim_ = ∑_hkl_ [1/(N − 1)] 1/2 ∑_i_ | I_i_(hkl) − [I(hkl)]|/∑_hkl_ ∑_i_ I_i_(hkl), where N is the redundancy for the hkl reflection

^c^R_work_/R_free_ = ∑_hkl_ | Fo − Fc |/∑_hkl_ | Fo |, where Fc is the calculated and Fo is the observed structure factor amplitude of reflection hkl for the working/free (5%) set

## Additional file

**Additional file 1.** Michaelis–Menten Kinetics and Lineweaver–Burk plots of Chit42 and the substrates analysed (**Figure S1**), mass spectrum data from the reaction mixtures obtained with colloidal chitin and chitosan QS1 as substrate (**Figure S2**, **Tables S1** and **S2**) as well as the HPAEC-PAD chromatograms of reactions obtained using different chitosans as substrates (**Figure S3**). **Figure S1.** Michaelis–Menten Kinetics of chitinase Chit42 expressed in *P. pastoris* and the substrates: colloidal chitin (a1), chitosan QS1 (b1), chitosan QS2 (c1), chitosan CHIT600 (d1), and chitosan CHIT100 (e1) at the indicated concentrations in mg/mL. Lineweaver–Bruk plots (double reciprocal plots) for the referred substrates are shown in the panels of the right column (a2, b2, c2, d2 and e2). V_max_ and *K*_m_ values (from Sigma Plot version 11) are also shown. **Figure S2.** Mass spectrum of the reaction mixtures obtained with colloidal chitin (a) and chitosan QS1 (b) as substrate. Reaction conditions: substrates 0.8% (w/v), 35 ºC, 24 h. Molecular masses plus sodium were detected in positive mode. The peaks corresponding to identified COS, by the availability of the corresponding standard, are indicated. **Figure S3.** Chromatograms of reactions obtained with different chitosans as substrate. HPAEC-PAD analysis of reactions based on the referred chitosans. Reaction conditions: 0.8% (w/v) of the chitosan indicated, 100 mM sodium acetate pH 5.5, 35 ºC, 24 h reaction. (1) GlcNAc; (2) (GlcNAc)2. The chromatogram of the indicated acetylated standards is also included. Other peaks could not be identified due to lack of the commercial corresponding standard.

## Abbreviations

COS: chitooligosaccharides
paCOS: partially acetylated COS
GlcN: d-glucosamine
GlcNAc: *N*-acetyl-β-d-glucosamine
GH: glycoside hydrolase
DP: degree of polymerization
DD: degree of deacetylation
PAGE: polyacrylamide gel electrophoresis
