# Supplementary material for: Use of chitin and chitosan to produce new chitooligosaccharides by chitinase Chit42: enzymatic activity and structural basis of protein specificity
Source: Microb Cell Fact. 2018 Mar 22;17:47. doi: 10.1186/s12934-018-0895-x (PMC5863366; doi:10.1186/s12934-018-0895-x)
Supplement: Supplementary file 1 — Additional file 1. Michaelis–Menten Kinetics and Lineweaver–Burk plots of Chit42 and the substrates analysed (Figure S1), mass spectrum data from the reaction mixtures obtained with colloidal chitin and chitosan QS1 as substrate (Figure S2, Tables S1 and S2) as well as the HPAEC-PAD chromatograms of reactions obtained using different chitosans as substrates (Figure S3). Figure S1. Michaelis–Menten Kinetics of chitinase Chit42 expressed in P. pastoris and the substrates: colloidal chitin (a1), chitosan QS1 (b1), chitosan QS2 (c1), chitosan CHIT600 (d1), and chitosan CHIT100 (e1) at the indicated concentrations in mg/mL. Lineweaver–Bruk plots (double reciprocal plots) for the referred substrates are shown in the panels of the right column (a2, b2, c2, d2 and e2). Vmax and Km values (from Sigma Plot version 11) are also shown. Figure S2. Mass spectrum of the reaction mixtures obtained with colloidal chitin (a) and chitosan QS1 (b) as substrate. Reaction conditions: substrates 0.8% (w/v), 35 ºC, 24 h. Molecular masses plus sodium were detected in positive mode. The peaks corresponding to identified COS, by the availability of the corresponding standard, are indicated. Figure S3. Chromatograms of reactions obtained with different chitosans as substrate. HPAEC-PAD analysis of reactions based on the referred chitosans. Reaction conditions: 0.8% (w/v) of the chitosan indicated, 100 mM sodium acetate pH 5.5, 35 ºC, 24 h reaction. (1) GlcNAc; (2) (GlcNAc)2. The chromatogram of the indicated acetylated standards is also included. Other peaks could not be identified due to lack of the commercial corresponding standard. [file 12934_2018_895_MOESM1_ESM.zip › new Table S2.docx]

**Table S2.** Main peaks and intensities of the mass spectrum corresponding to the reaction mixture obtained with chitosan QS1 as substrate and Chit42.

| **m/z** | **Intensity (counts)** | **% Intensity** |
| --- | --- | --- |
| 164.883468 | 351 | 3.5 |
| 230.934946 | 424 | 4.2 |
| 235.927486 | 153 | 1.5 |
| 244.077433 | 542 | 5.4 |
| 245.078954 | 86 | 0.9 |
| 260.072998 | 77 | 0.8 |
| 274.277029 | 352 | 3.5 |
| 290.276204 | 277 | 2.8 |
| 296.281901 | 110 | 1.1 |
| 304.325201 | 5983 | 59.7 |
| 318.314128 | 182 | 1.8 |
| 335.043193 | 212 | 2.1 |
| 340.963928 | 267 | 2.7 |
| 397.026361 | 547 | 5.5 |
| 405.190605 | 2339 | 23.3 |
| 406.190562 | 378 | 3.8 |
| 413.008934 | 234 | 2.3 |
| 481.026759 | 107.5 | 1.1 |
| 502.996226 | 331 | 3.3 |
| 516.991974 | 170 | 1.7 |
| 566.284322 | 10019 | 100.0 |
| 567.284045 | 2096 | 20.9 |
| 568.279987 | 460 | 4.6 |
| 569.034264 | 160 | 1.6 |
| 582.257008 | 1128 | 11.3 |
| 608.296517 | 5440 | 54.3 |
| 609.300677 | 1473 | 14.7 |
| 610.301716 | 272 | 2.7 |
| 624.272374 | 765 | 7.6 |
| 727.359862 | 572.5 | 5.7 |
| 728.362051 | 202.5 | 2.0 |
| 769.369786 | 1505.5 | 15.0 |
| 770.355817 | 518.5 | 5.2 |
| 771.367893 | 128.5 | 1.3 |
| 785.343885 | 306.5 | 3.1 |
| 814.646919 | 512 | 5.1 |
| 841.048471 | 148 | 1.5 |
| 846.417279 | 142 | 1.4 |
| 856.037482 | 79.5 | 0.8 |
| 857.01575 | 104.5 | 1.0 |
| 888.41741 | 296 | 3.0 |
| 889.444934 | 95 | 0.9 |
| 904.317605 | 226 | 2.3 |
| 930.437247 | 242 | 2.4 |
| 931.457647 | 96 | 1.0 |
| 946.297392 | 130 | 1.3 |
| 1007.50083 | 82 | 0.8 |
| 1049.48308 | 1877 | 18.7 |
| 1050.47892 | 655 | 6.5 |
| 1051.48154 | 201 | 2.0 |
| 1065.43508 | 213 | 2.1 |
| 1091.48096 | 171 | 1.7 |
| 1210.51422 | 100 | 1.0 |
| 1211.51676 | 49 | 0.5 |
| 1212.52002 | 22 | 0.2 |
| 1371.56196 | 72 | 0.7 |
| 1372.55689 | 32 | 0.3 |
| 1373.56802 | 31 | 0.3 |
| 1532.61349 | 131 | 1.3 |
| 1533.61344 | 88 | 0.9 |
| 1534.63293 | 34 | 0.3 |

[M+Na]^+^ peaks were detected in positive mode.
